# A Bill for an Act to Regulate the Practice of Dentistry in the State of Minnesota

**Published:** 1889-06

**Authors:** 


### A BILL.

### A Bill for an Act to Regulate the Practice of Dentistry in the State of Minnesota.

*Be it enacted by the Legislature of the State of Minnesota.*

SECTION 1. From and after September 1, 1889, it shall be unlawful for any person to practice dentistry in this State, unless he shall first have obtained a certificate of registration thereto, and filed the same or a certified copy thereof with the clerk of the district court of the county of his residence, all as hereinafter provided.

SEC. 2. A board of examiners to consist of five resident practicing dentists is hereby created, whose duty it shall be to carry out the purposes and enforce the provisions of this Act. The members of the first board under the provisions of this Act shall consist of the members of the present board of dental examiners, existing under chapter 199 of the General Laws of 1885, who shall hold their offices as members of such new board for the term for which they were appointed under said former act, and until their successors are duly appointed. All vacancies in said board shall be filled by appointment by the Governor as hereinafter provided. The term for which members of said board shall be appointed shall be three years, and until their successors shall be duly appointed. It is also hereby provided that no person shall serve to exceed two terms in succession. In case of any vacancy occurring in said board in the term of any member of said board, such vacancy shall be filled for such unexpired term by the Governor from names to be presented to him within two months of the occurrence of such vacancy by the Minnesota State Dental Association in the same manner as hereinafter provided.

It shall be the duty of said Minnesota State Dental Association after September 1, 1889, annually prior to August 10th to present to the Governor the names of twice as many practicing dentists resident in this State as there are regular members to be appointed of said board prior to September 1st, in the following year. All appointments by the Governor shall be made within twenty days of the submission of such names to him, and if such names shall not be submitted to him within the allotted time, he shall make his appointments within twenty days from the expiration of the time allotted for such presentation of names from among the resident practicing dentists. Provided, That nothing in this act shall prevent the appointment of two members of said board from among the resident practicing dentists not members of said Minnesota State Dental Association, if the Governor shall so elect.

SEC. 3. Said board shall choose at its first regular meeting, annually, one of its members president and one secretary thereof, who severally shall have the power during their term of office to administer oaths and take affidavits, certifying thereto under their hand and the seal of the said board. And after September 1, 1889, said board shall meet regularly at least twice in each year, to-wit: On the first Tuesday in April and October, and at such other times as may be deemed necessary by the board. Such meetings shall be held at the Medical Department of the University of the State of Minnesota. A majority of said board shall at all times constitute a quorum and the proceedings thereof shall at all reasonable times be open to public inspection. And it is furthermore provided, that in the event of any member of said board absenting himself from two of its regular meetings consecutively, the board shall declare a vacancy to exist, which vacancy shall be filled by the means hereinbefore provided.

SEC. 4. It shall be the duty of the first board hereinbefore provided for, to meet at the city of Duluth in said State on the second Tuesday in July, 1889, and elect officers, and within ten days thereafter to transfer to a register to be provided by them for that purpose, the name, residence, and place of business of each and every person who on the second Wednesday in July,

1889, and pursuant to an act of the Legislature of the State of Minnesota, approved March 3, 1885, shall be qualified to practice dentistry in the State of Minnesota, and who shall then be duly registered on the books of the board created by said act of March 3, 1885. No certificates of license to practice dentistry shall be issued after the second Wednesday in July, 1889, under said act of March 3, 1885. It shall be the duty of the said secretary of the first board hereby created to send to each person so registered prior to August 5, 1889, a certificate of his enregistration signed by the president and secretary of such board of examiners.

SEC. 5. Any person or persons who shall desire to begin the practice of dentistry in the State of Minnesota on and after September 1st, 1889, shall file his name, together with an application for examination with the Secretary of the State Board of Dental Examiners and at the time of making such application shall pay to the secretary of said board a fee of ten dollars, and shall present himself at the first regular meeting thereafter of said board to undergo examination before that body. In order to be eligible for such examination such person shall present to said board his diploma from some dental college in good standing, and shall give satisfactory evidence of his rightful possession of the same, provided also that the board may in its discretion admit to examination such other persons as shall give satisfactory evidence of having been engaged in the practice of dentistry ten years prior to the date of passage of this act. Said board shall have the power to determine the good standing of any college or colleges from which such diplomas may have been granted. The examinations shall be elementary and practical in character, but sufficiently thorough to test the fitness of the candidate to practice dentistry. It shall include, written in the Engtish language, questions on the following subjects: Anatomy, physiology, chemistry, materia medica, therapeutics, metallurgy, histology, pathology, operative and surgical dentistry, mechanical dentistry, and also demonstrations of their skill in operative and mechanical dentistry. All persons successfully passing such examinations shall be registered as licensed dentists in the board register provided for in Section 4, and also receive a certificate of such enregistration, said certificate to be signed by the president and secretary of the board. The examination fee shall in no case be refunded.

Sec. 6. Recipients of said certificate of enregistration shall present the same for record to the clerk of the district court of the county in which they reside, and shall pay a fee of fifty cents to said clerk for registration of the same. Said clerk shall record said certificate in a book to be provided by him for that purpose. Any person so licensed removing his residence from one county to another in this State, before engaging in the practice of dentistry in such other county shall obtain from the clerk of the district court of the county in which said certificate of registration is recorded a certified copy of such record or else obtain a new certificate of registration from the board of examiners, and shall, before commencing practice in such county, file the same for record with the clerk of the court of the county to which he removes, and pay the clerk for recording the same the fee of fifty cents. Any failure, neglect, or refusal on the part of any person holding such certificate or copy of record to file the same for record as hereinbefore provided, for six months from the issuance thereof shall forfeit the same. Such board shall be entitled to the fee of one dollar for the reissue of any certificate, and the clerk of the district court for any county shall be entitled to the fee of one dollar for making and certifying a copy of the record of any such certificate.

Sec. 7. All persons shall be said to be practicing dentistry within the meaning of this act, who shall for a fee or salary, or other reward paid either to himself or to another person for operations or parts of operations of any kind, treat diseases or lesions of the human teeth or jaws, or correct malpositions thereof. But nothing in this act contained shall be taken to apply to acts of bona fide students of dentistry done in the pursuit of clinical advantages under the direct supervision of a preceptor or a licensed dentist in this State, during the period of their enrollment in a dental college and attendance upon a regular uninterrupted course in such college.

SEC. 8. Out of funds coming into the possession of the board the members of said board may receive, as compensation, the sum of five dollars for each day actually engaged in the duties of their office, and mileage at three cents per mile for all distance necessarily traveled in going to and coming from meetings of the board. Said expenses shall be paid from the fees and assessments received by the board under the provisions of this act, and no part of the salary or other expenses of the board shall ever be paid out of the State Treasury. All moneys received in excess of said per diem allowance and mileage as above provided for shall be held by the secretary of said board as special fund for meeting expenses of said board and carrying out the provisions of this act, he giving such bond as the board shall from time to time direct. And said board shall make an annual report of its proceedings to the Governor by the 15th of December of each year, which report shall contain an account of all moneys received and disbursed by them pursuant to this act.

SEC. 9. Any person who shall violate any of the provisions of this act shall be deemed guilty of a misdemeanor, and upon conviction may be fined not less than twenty dollars nor more than one hundred dollars, or to be confined not less than one month nor more than three months in the county jail, or both. And all fines thus received shall be paid into the common school fund of the county in which such conviction takes place.

SEC. 10. Any person who shall knowingly or falsely claim or pretend to have or hold a certificate of enregistration, diploma, or degree granted by a society or by said board, or who shall falsely and with intent to deceive the public, claim or pretend to be a graduate from any incorporated dental college, not being such graduate shall be deemed guilty of a misdemeanor, and shall be liable to the penalties provided for in section nine of this act.

SEC. 11. Justices of the peace and the respective municipal courts shall have jurisdiction over violations of this act. It shall be the duty of the respective county attorneys to prosecute all violations of this act.

SEC. 12. Any person who shall be licensed under the provisions of this act, and who shall practice dentistry under a false name with intent to deceive the public, shall be liable to have said license revoked upon twenty days notice of such proposed revocation, and of the time and place of considering such revocation by order of the State Board of Dental Examiners. And any person, who, after revocation of his license shall continue to practice dentistry in the State of Minnesota shall be deemed guilty of a violation of the provisions of this act and shall be subject to the penalties provided therein. Nor shall a certificate to a person under one name by any defense to an action brought against him for practicing without a certificate under another, unless it be shown that such practice under such other name was done without intent to defraud or deceive.

SEC. 13. Every registered dentist shall in each and every year after 1889 pay to said board of examiners the sum of one dollar as a license fee for such year. Such payment shall be made prior to May 1st in each and every year, and in case of default of such payment by any person, his certificate may be revoked by the board of examiners upon twenty days notice of the time and place considering such revocation. But no license shall be revoked for such non-payment if the person so notified shall pay before or at such consideration his fee, and such penalty as may be imposed by said board, provided that said board may impose a penalty of five dollars and no more on any one so notified as a condition of allowing his license to stand. Provided further that said board of examiners may collect such dues by suit.

SEC. 14. The board of examiners created by this act may sue or be sued, and in all actions brought by or against it, it shall be made a party under the name of the Board of Dental Examiners of the State of Minnesota. And no suit shall abate by reason of any change in the membership of said board.

SEC. 15. Chapter 199 of the General Laws of 1885, being an act entitled "An Act to Insure the Better Education of the Practitioners of Dental Surgery, and to Regulate the Practice of Dentistry in the State of Minnesota," approved March 3d, 1885, is hereby repealed such repeal to take effect September 1st, 1889.

SEC. 16. All effects and property whatsoever of the board of dental examiners created by said act of March 3d, 1885, shall on said first day of September, 1889, be and become the property of the board of examiners created by this act, and said board hereby created is hereby declared to be the legal successor of the board created by said act of March 3d, 1882.

SEC. 17. This act shall take effect and be in force from and after its passage.

*Approved April 24th*, 1889.

